# Formononetin ameliorates SP-induced urticaria in mice via suppressing TAK1/MAK signaling pathway

**DOI:** 10.1371/journal.pone.0340078

**Published:** 2026-01-23

**Authors:** Yan Wu, Chunyu Li

**Affiliations:** Rehabilitation and Health Department, Anhui College of Traditional Chinese Medicine, Wuhu, Anhui, China; Indian Institute of Technology Delhi, INDIA

## Abstract

**Background:**

Chronic idiopathic urticaria (CIU) is a condition that significantly impacts patient well-being, requiring effective therapeutic strategies. Formononetin, a natural isoflavone with anti-inflammatory properties, has shown promise in allergic conditions. However, its specific effects and mechanism in CIU are not fully understood.

**Methods:**

Comparative analyses were conducted between normal and formononetin-treated groups, along with mechanistic investigations into the TAK1/MAPK pathway both *in vivo* and *in vitro*. Cell morphology, cytokine secretion, histamine release, and TAK1/MAPK pathway alterations were assessed.

**Results:**

Formononetin treatment led to a dose-dependent reduction in MC degranulation, histamine release, and secretion of inflammatory cytokines (TNF-α, IL-1β, IL-6). Additionally, formononetin inhibited the phosphorylation of TAK1, p38, ERK1, and JNK similar to a TAK1 inhibitor in murine and cellular models.

**Conclusion:**

Formononetin shows potential as an anti-allergic agent by alleviating inflammatory responses in CIU through suppression of the TAK1/MAPK pathway in both murine models and MC/9 cells.

## 1. Introduction

Chronic idiopathic urticaria (CIU) is a prevalent allergic disorder that afflicts a considerable number of patients, characterized by the recurring appearance of itchy, red welts or raised bumps on the skin, also including swelling, a burning sensation, or even pain, and recurrent episodes of the condition that persist for over six weeks [1]. It significantly impairs quality of life in patients and may even induce psychological comorbidities, such as anxiety and depression [2]. Despite the availability of various treatment modalities, including antihistamines, corticosteroids, and immunomodulators, managing CIU remains challenging due to suboptimal efficacy, adverse effects, and the lack of long-term remission in many cases [3]. Moreover, a considerable proportion of patients exhibit inadequate response or intolerance to conventional therapies, highlighting the urgent need for novel therapeutic approaches. Therefore, there is a pressing demand for the development of effective and well-tolerated pharmacological interventions to alleviate the symptoms and improve the outcomes of CIU. In this context, exploring natural compounds with potential anti-urticarial properties represents a promising avenue for the identification of novel therapeutic agents.

Mast cells (MCs), sentinel immune cells that play a pivotal role in allergic diseases, have been identified as the effector cells in the establishment of CIU. The exposure to external allergens triggers cross-linking of immunoglobulin (Ig) E antibodies, then IgE receptor (FcεR) aggregate and undergo IgE-induced MCs degranulation, resulting in the release of histamine and other proinflammatory factors, which has been employed to assess the severity in patients with CIU and to specifically evaluate mast cell degranulation [4,5].

Antigen (Ag)-dependent cross-linking of multiple IgE-FcεRI complexes *in vitro* activate several important signaling cascades including the TAK1 (Transforming growth factor-β (TGF-β)-activated kinase 1), MAPK (Mitogen-activated protein kinase), NF-κB (Nuclear factor kappa B), and phospholipase C γ pathways among others occurring *in vivo* [6]. TAK1 has been characterized as an important kinase in activated MCs that triggers degranulation and cytokine responses in allergic inflammation [7]. For MC-dependent inflammatory reactions, TAK1 and its binding protein TAB transmit signals to activate the MAPK cascades and the IKK complex, thereby promoting the MAPK and NF-κB pathways, leading to production of IL-1β (interleukin-1β), TNF-α(tumor necrosis factor), and IL-6 (interleukin), among others [8]. Studies have also shown that a later increase of IL-1β, TNF-α, and IL-6 mRNA levels follows the activation of three different MAPKs (p38, ERK, JNK), consistent with a possible relationship between MAPK and pro-inflammatory cytokine expression [9]. A recent study revealed that the upstream kinase TAK1/MAPK pathway was correlated with the initiation of MCs degranulation in the early-phase response to allergen recognition and inflammation in the late-phase response [7], suggesting the effect of TAK1/MAPK pathway on pathogenesis in allergic diseases. However, there remains limited research focusing TAK1/MAPK pathway on CIU pathogenesis.

Formononetin (7-hydroxy, 4′-methoxyisoflavone) is a natural isoflavone that can be extracted from many medicinal plants, including *Pongamia pinnata* [10], *Astragalus membranaceus*, [11] *Ononis angustissima* [12] and *Trifolium pretense* [13]. Studies have investigated various pharmacological effects of formononetin, such as anticancer, antimicrobial and antiviral, antioxidant, also anti-inflammatory properties [14,15]. Recent researches have highlighted the significant anti-inflammatory properties of plants rich in isoflavones in combating allergic conditions like dermatitis and respiratory issues among other pharmacological activities [16]. Study has shown that formononetin restores epithelial barrier function by inhibiting cytokines derived from epithelial cells and producing thymic stromal lymphopoietin in a mouse model with atopic contact dermatitis, exhibiting strong potential for alleviating allergic diseases [14]. Formononetin was also well-pronounced in inhibiting histamine release and secretion of inflammation-related factors, including TNF-α, IL-1β and IL-6, indicating formononetin as a potential molecule in suppressing allergic inflammatory response. However, it has not yet been applied to treat allergic diseases, indicating insufficient studies on revealing the pathological mechanism and effects of formononetin treatment on allergic diseases, such as chronic urticaria.

Herein, we hypothesize formononetin alleviates CIU inflammation and involves the TAK1/MAPK pathway in the mechanism. This study is dedicated to clear out the anti-allergic inflammatory effects of formononetin and its possible regulating mechanism on CIU.

## 2. Materials and methods

### 2.1. Animals

Thirty healthy male BALB/c mice (6 weeks old) were purchased from Southern Medical University (Guangzhou, China). All mice were placed on a regular granular diet and given free access to sterilized tap water and food. In addition, the mice were stored in a controlled environment with the following conditions: temperature, 18−22 ˚C; relative humidity, 40−70%; noise, < 50 dB; and 12-h light/dark cycle. This study was conducted in strict adherence to the recommendations of the ARRIVE guidelines and received approval from the Institutional Animal Care and Use Committee of Traditional Chinese Medicine Hospital in Wuhu City (NO. YW2024−017). Efforts were made to minimize animal suffering throughout the study. Mice were anesthetized with pentobarbital sodium (50 mg/kg, intraperitoneally) before all invasive procedures, and euthanasia was performed by cervical dislocation under deep anesthesia at the end of the experiment.

### 2.2. Mouse model of CIU, treatment and grouping

Given the complex and diverse epitology of CIU patients and the not fully-understood mechanism of pathologensis, it has been challenging to identify the external pathogenic factors. Mas-related G protein-coupled receptor X2 (MRGPRX2) that expresses on MCs has shown the pivotal impact on pseudoallergic reactions and is investigated to be activated by secretagogues, including substance P (SP) which has been successfully employed to induce chronic urticaria-like animal model. [17] Therefore, this experiment constructed an SP-induced CIU model and the successful establishment of model is evaluated by the degree of MCs infiltration and the release levels of histamine and cytokines according to the method of type I allergic chronic urticaria model.

Balb/c mice were randomly divided into 6 groups with 5 mice in each group (model group, treatment groups and normal group, and takinib group). All mice were depilated on the dorsal skin one day before the first subcutaneous injection. The model group received sensitization through subcutaneous injection of 0.5 mL of SP (30 μg/mL) at four time points—days 0, 7, 14, and 21—at four dorsal sites (0.125 mL per site). Following the same schedule, the formononetin treatment groups received intragastric administration of formononetin at doses of 5 mg/kg, 10 mg/kg, or 20 mg/kg once daily for 21 days, and 1 hour after each administration on days 0, 7, 14, and 21, the mice were given subcutaneous injections of 0.5 mL SP (30 μg/mL) as described above. In contrast, the normal group received saline via gavage, followed by subcutaneous injection of 0.5 mL of saline at four dorsal sites 1 hour later on the same days.

The takinib group received takinib (50 mg/kg) by intragastric administration once daily for 21 days, following the same schedule as the formononetin treatment groups, and 1 hour after each administration on days 0, 7, 14, and 21, subcutaneous injections of 0.5 mL SP (30 μg/mL) were performed. This ensured consistent pre-treatment and comparable treatment procedures between formononetin- and takinib-treated animals [18].

On day 25, mice in the model, formononetin, and takinib groups were challenged via tail-vein injection with 0.2 mL SP (30 μg/mL), while those in the normal group received 0.2 mL of saline. Animals were euthanized 3 hours after the final injection, and both skin tissue and whole blood samples were collected for subsequent analyses.

### 2.3. Hematoxylin/eosin (H&E) and toluidine blue staining

The skin tissues were made paraffin slices using slicer (Leica, RM2016). For H&E staining, paraffin sections were routinely dewaxed in water, then stained with hematoxylin for 10 min, and then treated for 10 s with 1% hydrochloric acid and ethanol. After dyeing with eosin for 5 min, the sample was dehydrated with gradient ethanol, clarified in xylene, and sealed with neutral balm. For toluidine blue staining, paraffin sections were dewaxed and dehydrated and stained with 1% toluidine blue for 20 min, washed with ddH_2_O to remove the excess dye, then gradually dehydrated in different concentrations of alcohol (70%–95%) and transparent with xylene, and sealed with neutral resin after drying. The images were observed and analyzed using an inverted fluorescence microscope (NIKON, DS-U3, Japan).

### 2.4. Mast cell culture, treatment and activation

MC/9 mast cell was obtained from Guangzhou Jennio Biotech Co., Ltd. (Guangzhou, China) and were cultured in DMEM with 10% fetal bovine serum, 100 U/ml penicillin and 100 µg/ml streptomycin at 37˚C in a humidified 5% CO_2_ atmosphere, and maintained at a density of 0.25 × 10^6^ cells/ml and media was replaced every 3–4 days. Cells were divided into a control group and experimental groups. the control group were maintained under the normal aforementioned conditions, while cells in experimental groups were sensitized with anti-DNP IgE (10 µg/ml) for 16 h and pretreated with varying concentrations of formononetin (0µM, 0.1µM, 1µM and 10 µM) prior to treatment with DNP-HSA (anti-dinitrophenyl-human serum albumin) (500 ng/ml).

### 2.5. ELISA

Enzyme-linked immunosorbent assay method (ELISA) was conducted to measured cytokine concentrations of TNF-α, IL-1β, IL-6, and histamine from mice serum and cell culture supernatants of MC/9 cells according to the manufacturer’s protocol (OptEIA, Phamingen). The absorbance of each sample was recorded at 450 nm using a microplate reader (Thermo Fisher, Multiskan FC, USA).

### 2.6. CCK-8 assay of cell viability

Cell viability was determined using Cell Counting Kit-8 (CCK-8) assay. MC/9 cells were plateded in 96-well plates (5 × 10^4^ cells/well, 100 μL per well) and treated with 10 μL formononetin at varying concentrations (0μM, 0.1μM, 1μM, 10μM and 100 μM) for 24 h. After that, CCK-8 solution (10 μL) was added to each well, followed by incubation for 2 h. Cell viability was assessed by measuring absorbance at 450 nm using a microplate reader (Bio-Rad, Carlsbad, CA, USA).

### 2.7. Western blot analysis

After determination of total protein using a BCA kit (A045-4; Nanjing Jiancheng Bioengineering Institute). The collected protein extracts were separated using SDS-PAGE (10% gel), then transferred onto polyvinylidene fluoride membranes, and blocked for 2 h at room temperature by constant stirring in 5% non-fat milk in TBST. The expression of phosphorylated TAK1 (p-TAK1), TAK1, phosphorylated P38 (p-P38), P38, phosphorylated ERK (p-ERK), ERK, phosphorylated JNK (p-JNK) and JNK was detected using following primary antibodies (Cell Signaling Technology) incubated at 4˚C overnight: anti-GAPDH (#2118,1:2000), anti-p-TAK1 (#6943, 1:1000), anti-TAK1 (#4023, 1:1000), anti-p-p38 (#2731, 1:1000), anti-p38 (#2796, 1:1000), anti-p-Erk1/2 (#9101, 1:1000), anti-Erk1/2 (#9102, 1:1000), anti-p-JNK (#4060, 1:1000), anti-JNK (#4691, 1:1000). The membranes were incubated with secondary antibodies (1:20,000 in TBST) for 1 h at 37 ◦C after washed thrice with TBST, after which they were developed using an enhanced chemiluminescence kit. Finally, protein levels were visualized by enhanced chemiluminescence (ECL) kit and analyzed using Image Pro Plus 5.1 software.

### 2.8. Statistical analysis

Data are expressed as the mean ± standard deviation. GraphPad Prism 5.0 (GraphPad Software, Inc., La Jolla, CA, USA) and SPSS22.0 (SPSS Inc., Chicago, IL, USA) were used for statistical analysis. The experimental data from different groups were analyzed by one-way analysis of variance (ANOVA) followed by a Tukey’s test for multiple comparisons. Differences were considered statistically significant when p < 0.05.

## 3. Result

### 3.1. Formononetin ameliorates MCs degranulation and inflammation in CIU mice

**Fig 1:**
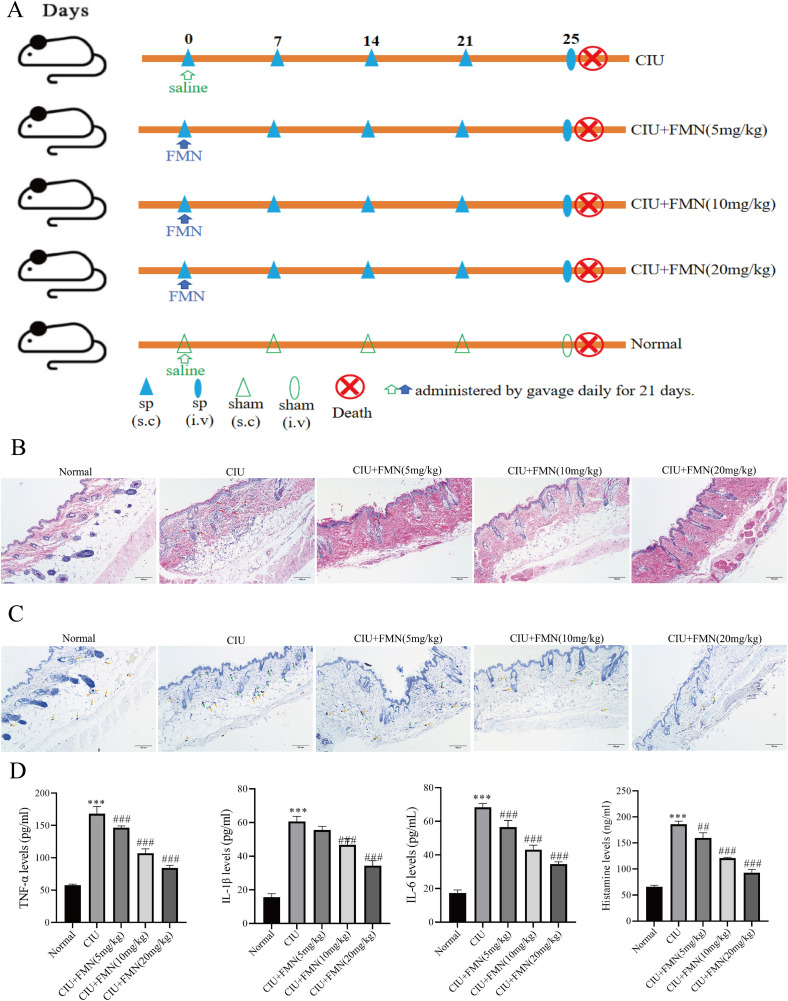
Formononetin ameliorates MCs degranulation and inflammation in CIU mice. The effect of formononetin on CIU. **(A)** The abridged general view of SP induced CIU mouse model construction. **(B-C)** The representative pictures of eosinophil and MCs degranulation on skin response under formononetin pretreatment in SP-induced CIU mice detected by H&E staining (×100) and toluidine blue (TB) staining (×100). **(D)** The effect of formononetin pretreatment on SP-induced serum concentrations of TNF-α, IL-1β, IL-6, and histamine measured by ELISA. Measurement data are expressed as the mean ± SDM (n = 5). One-way ANOVA was used to determine significance in statistical comparisons. Statistical significance was defined at ^***^ p < 0.001 vs. the normal group; ^##^ p < 0.01, and ^###^ p < 0.001 vs. the CIU model group. FMN, formononetin. Specifically, red arrows indicate eosinophils, yellow arrows mark mast cells, and green arrows highlight degranulation sites.

We established an SP-induced CIU model in mice to evaluate the effect of formononetin on CIU (Fig 1A). In comparison to healthy mice, CIU mice displayed a more prominent presence of degranulated MCs, characterized by pronounced swelling and deformation, irregular cell edges, and compromised membrane integrity, resulting in granule detachment (Fig 1B, C). These observed differences between the model and normal groups, as indicated by subsequent pathological assessments, affirming the successful establishment of the CIU model. Following the increasing dose of treatment, HE staining of mouse skin showed that formononetin inhibited vasodilation and mitigated the release of eosinophils (Fig 1B and S1A Fig). Toluidine blue staining of corresponding back skin sections demonstrated a reduction in the number of degranulated MCs under formononetin treatment (Fig 1C and S1B-C Fig). In addition, the serum concentrations of TNF-α, IL-1β, IL-6, and histamine significantly decreased by formononetin pretreatment (Fig 1D). These findings indicates that formononetin could reduce the symptoms of SP-induced chronic urticaria via suppressing MCs allergic response in mice.

### 3.2. The anti-allergic effects of formononetin in DNP-HSA–stimulated MC/9 cells

**Fig 2:**
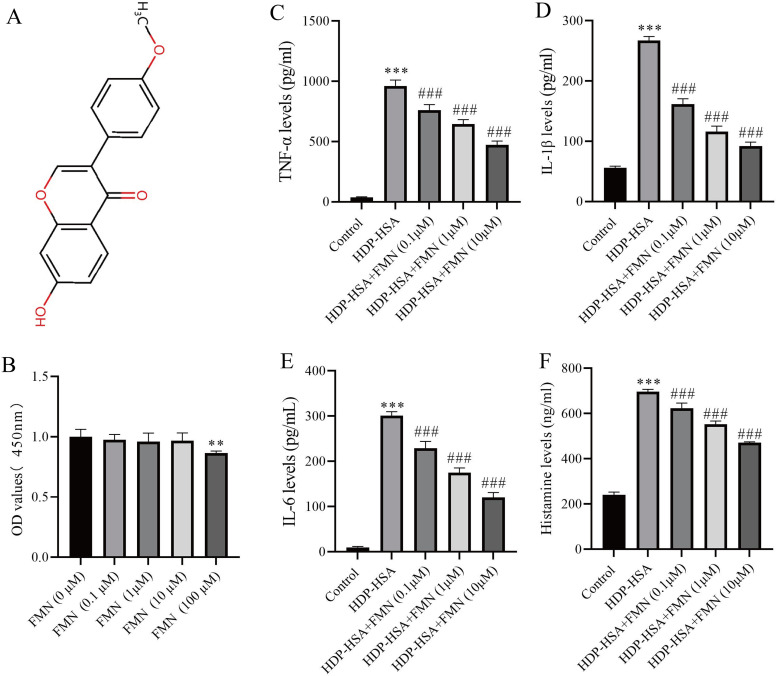
The effects of formononetin on cytotoxicity and the secretion of cytokines and histamine in MC/9 cells. **(A)** Chemical structure of formononetin. **(B)** The viability of MC/9 cells treated with formononetin. **(C-F)** The inhibitory effect of formononetin on the secretion of TNF-α **(C)**, IL-1β **(D)**, IL-6 **(E)**, and histamine (F) in MC/9 cells stimulated with DNP-HSA (16 hours) and unstimulated counterparts. Measurement data are expressed as the mean ± SDM (n = 5). One-way ANOVA were used to determine significance in statistical comparisons. Statistical significance was defined at ^***^ p < 0.001 vs. the control group; ^##^ p < 0.01, and ^###^ p < 0.001 vs. the DNP-HSA-stimulated group.

CCK-8 assays of the cell viability assay showed that formononetin treatment (0.1–10 µM) had minor toxic effects on MC/9 cells (Fig 2B). Furthermore, the secretion of the pro‑inflammatory cytokines TNF-α, IL-1β, IL-6, and histamine was determined to evaluate the effect of formononetin on inflammation (Fig 2C-F). Compared to the control group, the secretions of TNF-α, IL-1β, IL-6 and histamine in MC/9 cells stimulated with DNP-HSA, an established *in vitro* model for IgE-mediated allergic mast cell activation, was significantly increased (all p < 0.001; Fig 2C-F). Following formononetin treatment, the levels of TNF-α, IL-1β, IL-6, and histamine were significantly and dose-dependently reduced in DNP-HSA-stimulated MC/9 cells compared with the DNP-HSA-only group (0.1 µM, p < 0.05; 1 µM, p < 0.01; 10 µM, p < 0.001; Fig 2C–F). These findings indicate that formononetin significantly reduces allergic inflammatory mediator release in IgE-mediated mast cell activation, suggesting its potential therapeutic role in attenuating allergic inflammation.

### 3.3. Formononetin acts on TAK1/MAPK pathway

**Fig 3:**
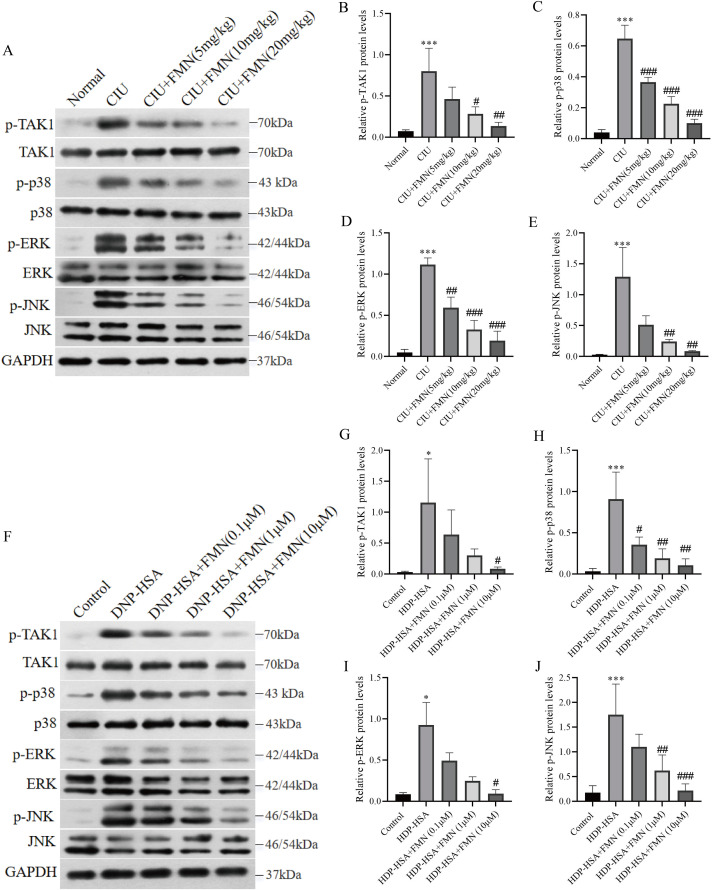
Formononetin suppresses the activation of TAK1/MAPK pathway. **(A)** A decrease in the phosphorylation of TAK1, ERK, JNK, and p38 was observed via western blot assay *in vivo*. **(B-E)** The densitometric analysis of western blots quantitatively confirmed this decrease in the SP-induced CIU model. **(F)** *In vitro* experiments (MC/9 cells) also revealed a decrease in the phosphorylation levels of TAK1, ERK, JNK, and p38 as observed via western blot assay. **(G-J)** Densitometric analysis of western blots further validated this reduction in phosphorylation levels in DNP-HSA-stimulated MC/9 cells. Measurement data are expressed as the mean relative phosphorylation derived from the same samples (phospho/total) ± SDM (n = 5). One-way ANOVA were used to determine significance in statistical comparisons. Statistical significance was defined at ^*^p < 0.05, ^**^p < 0.01, ^***^p < 0.001 vs. the normal or control group; ^##^p < 0.01, and ^###^p < 0.001 vs. the untreated model groups.

To further elucidate the mechanism underlying formononetin’s inhibitory effect on MCs degranulation, we conducted a comparative analysis of the TAK1/MAPK pathways between treatment and control groups, as well as among different treatment groups. This analysis was carried out in both the SP-induced CIU model and DNP-HAS-stimulated MC/9 cells using western blotting. In the mouse model, qualitative assessment of membrane images revealed a noticeable inhibition in the phosphorylation of all four major nodes in the TAK1/MAPK pathways—TAK1, ERK, JNK, and p38—in the treatment group compared to the untreated group (Fig 3A). This inhibitory effect was dose-dependent, as evidenced by subsequent quantitative measurements, which further confirmed a significant reduction in the phosphorylation levels of TAK1, ERK, JNK, and p38 with increasing doses of formononetin (Fig 3B-E). Moreover, the consistent inhibitory effect of formononetin was observed in DNP-HAS-stimulated MC/9 cells that the expression of p-TAK1, p-ERK, p-JNK, and p-p38 was decreased qualitatively and quantitatively which is less pronounced (0.1μM, 1μM, 10μM) than results in animal model yet still significantly impacted at 10μM formononetin treatment overall (Fig 3F-J). These findings from both *in vivo* and *in vitro* models collectively suggests that formononetin plays a crucial role in suppressing the activation of the TAK1/MAPK pathway.

### 3.4. Formononetin ameliorates CIU by suppressing TAK1/MAPK signaling, similar to the TAK1 inhibitor takinib

**Fig 4:**
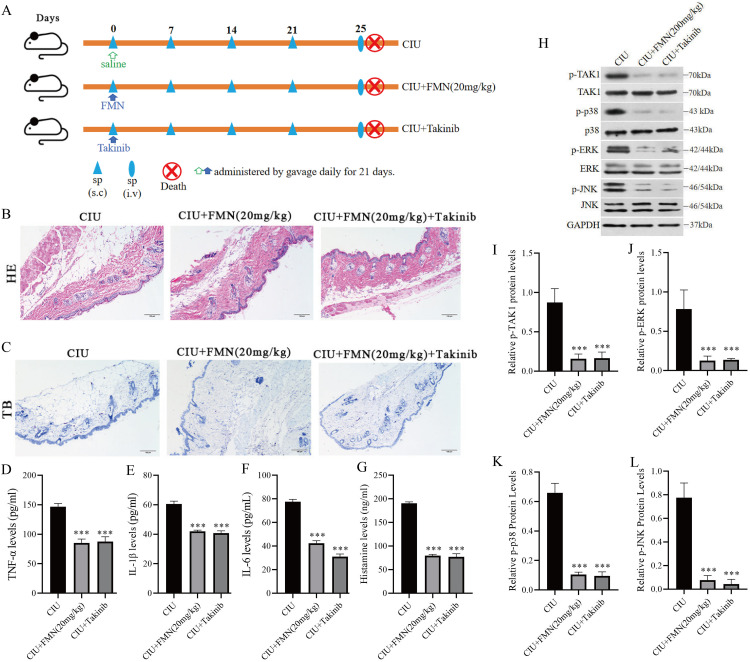
Formononetin decreased TAK1/MAPK signaling pathway in CIU as TAK1 inhibitor. **(A)** The abridged general view of SP induced CIU mouse model construction. **(B, C)** The pathological effects of formononetin on SP-induced skin response. And the consistent inhibition of formononetin and takinib on concentrations of TNF-α **(D)**, IL-1β **(E)**, IL-6 **(F)**, and histamine **(G)**. The consistent suppression of formononetin and takinib on TAK1/MAPK signal modulation in SP-induced CIU model was carried out by western blotting (H) and the corresponding quantitative analysis of p-TAK1 **(I)**, p-p38 **(J)**, p-ERK **(K)**, p-JNK **(L)**. Measurement data are expressed as the mean ± SDM (n = 5). One-way ANOVA were used to determine significance in statistical comparisons. Statistical significance was defined at ^***^p < 0.001 vs. the CIU model group.

Analyses of signaling pathways revealed that formononetin suppresses TAK1 signaling both *in vivo* and *in vitro*. To test the possibility that inhibition of TAK1 is the underlying mechanism behind the therapeutic effect of formononetin on SP-induced urticaria, we parallelly compared the effects of formononetin and the TAK1 inhibitor takinib on the phenotype and inflammatory responses in SP-induced CIU mice. Given that TAK1 is an upstream regulator in the TAK1/MAPK pathway, SP-induced CIU mice were treated with the TAK1 inhibitor (takinib, 50 mg/kg) to further determine whether formononetin alleviates CIU through the inhibition of TAK1 activation (Fig 4A). Histological evaluation using H&E and toluidine blue staining revealed that both formononetin treatment and the combined administration of formononetin and takinib significantly mitigated vasodilation, eosinophil infiltration, and MC degranulation in dorsal skin tissues compared with the model group (Fig 4B, C). Moreover, the serum concentrations of TNF-α (Fig 4D), IL-1β (Fig 4E), IL-6 (Fig 4F), and histamine (Fig 4G) were markedly reduced (p < 0.001) in both the formononetin and takinib groups compared to the CIU model group. Consistent with these findings, western blot analysis demonstrated that formononetin (20 mg/kg) significantly inhibited the phosphorylation of TAK1, ERK, JNK, and p38, with a suppression pattern comparable to that of takinib, both qualitatively (Fig 4H) and quantitatively (Fig 4I–L). These results suggest that formononetin alleviates SP-induced urticaria symptoms by inactivating the TAK1/MAPK signaling pathway, exhibiting a TAK1 inhibitory effect similar to that of takinib.

### 3.5. Formononetin suppresses mast cell activation via inhibition of the TAK1/MAPK pathway *in vitro*

**Fig 5:**
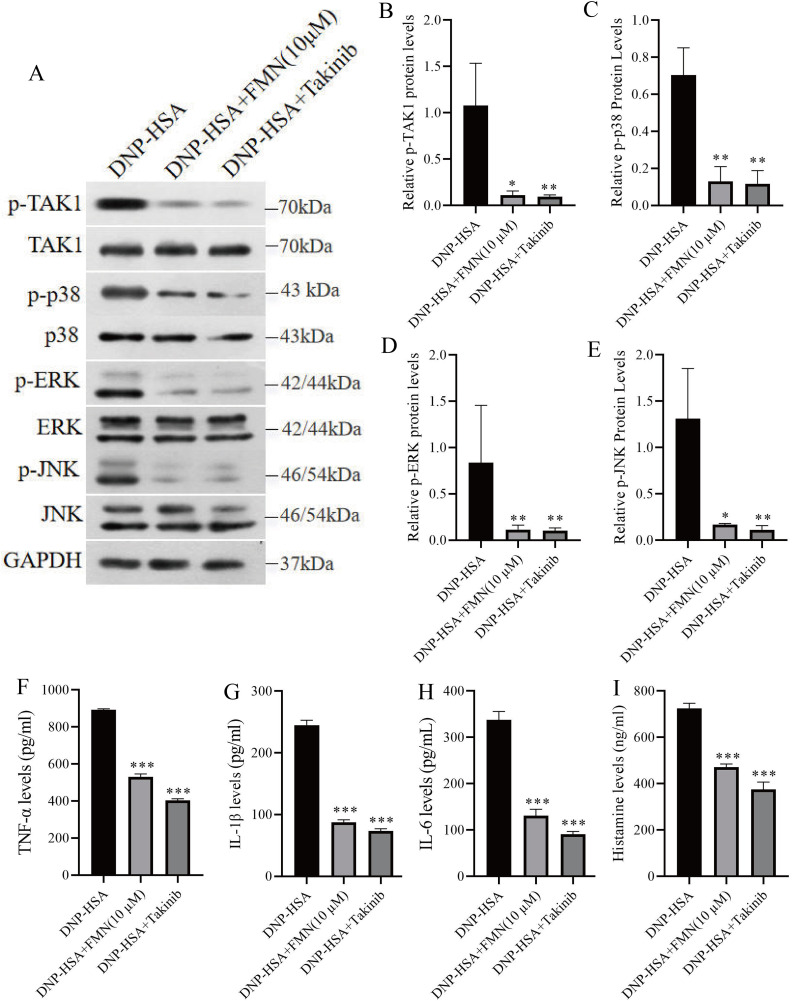
Formononetin decreased TAK1/MAPK signaling pathway in activated MC/9 cells. **(A-E)** Western blot analysis of p-TAK1, p-P38, p-ERK, p-JNK, TAK1, P38, ERK, JNK protein expression on DNP-HSA stimulated MC/9 treated with formononetin or takinib. (H-I) Quantative analysis of TNF-α, IL-1β, IL-6, and histamine by ELSA. Measurement data are expressed as the mean ± SDM. One-way ANOVA were used to determine significance in statistical comparisons. Statistical significance was defined at ^*^p < 0.05, ^**^p < 0.01, ^***^p < 0.001 vs. the untreated DNP-HAS stimulated cells.

To further validate whether formononetin exerts its anti-allergic effects by inhibiting TAK1 signaling in mast cells, we compared the effects of formononetin and the TAK1 inhibitor takinib (10 µM) in DNP-HSA-stimulated MC/9 cells. Western blot analysis revealed that both formononetin and takinib significantly reduced the phosphorylation of TAK1, p38, ERK, and JNK compared with untreated activated cells (Fig 5A–E). Furthermore, the secretion levels of TNF-α, IL-1β, IL-6, and histamine in cell supernatants were markedly decreased following treatment with formononetin or takinib (Fig 5F–I). These findings demonstrate that formononetin suppresses mast cell activation through inhibition of the TAK1/p38/ERK/JNK signaling cascade, consistent with the mechanism of TAK1 inhibition. Collectively, both *in vivo* and *in vitro* results support that formononetin mitigates allergic inflammation by acting as a functional inhibitor of the TAK1/MAPK pathway.

## 4. Discussion

The current study investigated the anti-allergic inflammatory effects of formononetin and its mechanism of action both *in vivo* and *in vitro*. SP is a neuropeptide that is a mediator of itch signaling and plays a key role in the regulation of inflammation in immunity [19]. Injecting SP into mice effectively induces MCs degranulation and histamine release, leading to the development of urticaria-like symptoms [20]. Therefore, SP was selected to construct CIU mice model to replicate overall symptoms and pathological changes controlled by formononetin *in vivo*. Moreover, CIU is mediated by MCs degranulation and causes allergic responses via inflammation [18]. MC/9 cells have been identified as a suitable cell line to conduct investigations into allergic inflammation *in vitro* [21]. Therefore, this study employed MC/9 cells stimulated by DNP-HSA to investigate the specific mechanism of formononetin acting on MCs.

Histamine is contained in the granules of MCs and is released when MCs degranulation in response to external allergens and IgE antibodies, which has been observed in RBL-2H3, BMMC, HMC-1 and LAD2 mast cell lines [22]. Following treatment with formononetin, the increased level of histamine in both SP-induce CIU mice and DNP-HSA stimulated MC/9 cells was reversed in a dose-dependent manner. These results indicate that formononetin alleviates CIU. TNF-α, IL-1β and IL-6 are effective biomarkers of CIU, and inhibition of the secretion of these proinflammatory cytokines effectively moderates inflammatory symptoms. The current study showed that formononetin significantly reduced the secretion of TNF-α, IL-1β and IL-6 in both SP-induced CIU mice and DNP-HSA stimulated MC/9 cells.

TAK1, a member of the mitogen-activated protein kinase kinase kinase (MAP3K) family, has been shown as a kinase for the MAPK pathway [23]. MAPK pathways were well-recognized for mediating MCs activation, survival, differentiation, and cytokine production [24]. The major nodes in MAPK pathway, ERK, JNK, and p38 affect AP-1 transcriptional activity, and thereby regulating the production of proinflammatory factors [25]. The significance of MAPK pathway in anti-inflammatory therapies has been highlighted in previous works [7]. For example, p38, a key member of the MAPK pathway, was demonstrated to modulate the production of IL-4 with its upstream kinase MKK3 in MCs activated by allergens [26]. Focusing on TAK1/MAPK pathway, the current study further demonstrated that formononetin suppresses the phosphorylation of TAK1, p38, ERK, and JNK in the TAK1/MAPK pathway in our constructed models, thereby alleviating allergic inflammation of CIU. Together with the important role that TAK1 plays in the TAK1/MAPK pathway, TAK1 inhibitor (takinib) was used in the constructed CIU mice and activated MC/9 cells, and revealed a comparable effect to formononetin, indicating formononetin may control the TAK1/MAPK pathway as a TAK1 inhibitor. However, this requires further experimental confirmation.

Formononetin, an isoflavonoid extracted from plants, has been recommended as a potential candidate drug for allergic responses and has become a subject of interest in various industries, particularly in human health and medicine [27]. In alleviating neuroinflammation, formononetin has been demonstrated to target NF-κB activity by inhibiting the phosphorylation of IKKα, and inhibit p-38, JNK and ERK activation by targeting upstream proteins TAK1 [28,29]. Nevertheless, no study to date reported its effect and possible mechanism on alleviating CIU. While this study demonstrated that formononetin mitigates CIU and attenuates MCs degranulation by reducing histamine release and inflammatory response through suppressing TAK1/MAPK pathway as a TAK1 inhibitor. These findings provide initial mechanistic insight that formononetin may alleviate the symptoms of CIU, expanding the potential application of formononetin in anti-allergic effects and contributing to treatment prospective of CIU.

In conclusion, this study demonstrated that formononetin exerts significant anti-allergic inflammatory effects both *in vivo* and *in vitro*. By using SP-induced CIU mice models and DNP-HSA stimulated MC/9 cells, it was shown that formononetin effectively reduces histamine release and the secretion of proinflammatory cytokines TNF-α, IL-1β, and IL-6. The underlying mechanism involves the inhibition of the TAK1/MAPK pathway, as evidenced by the suppression of TAK1, p38, ERK, and JNK phosphorylation. These findings suggest that formononetin may act as a TAK1 inhibitor, providing a novel therapeutic approach for alleviating CIU symptoms. Further research is needed to confirm the precise molecular mechanisms and to explore the potential clinical applications of formononetin in treating allergic inflammation.

## Supporting information

S1 Fig Formononetin ameliorates MCs degranulation and inflammation in CIU mice. (DOCX)

S2 Fig Original western blot images. (PDF)
