# Supplementary material for: Formononetin ameliorates SP-induced urticaria in mice via suppressing TAK1/MAK signaling pathway
Source: PLoS One. 2026 Jan 23;21(1):e0340078. doi: 10.1371/journal.pone.0340078 (PMC12829854; doi:10.1371/journal.pone.0340078)
Supplement: S1 Fig — (DOCX) [file pone.0340078.s001.docx]

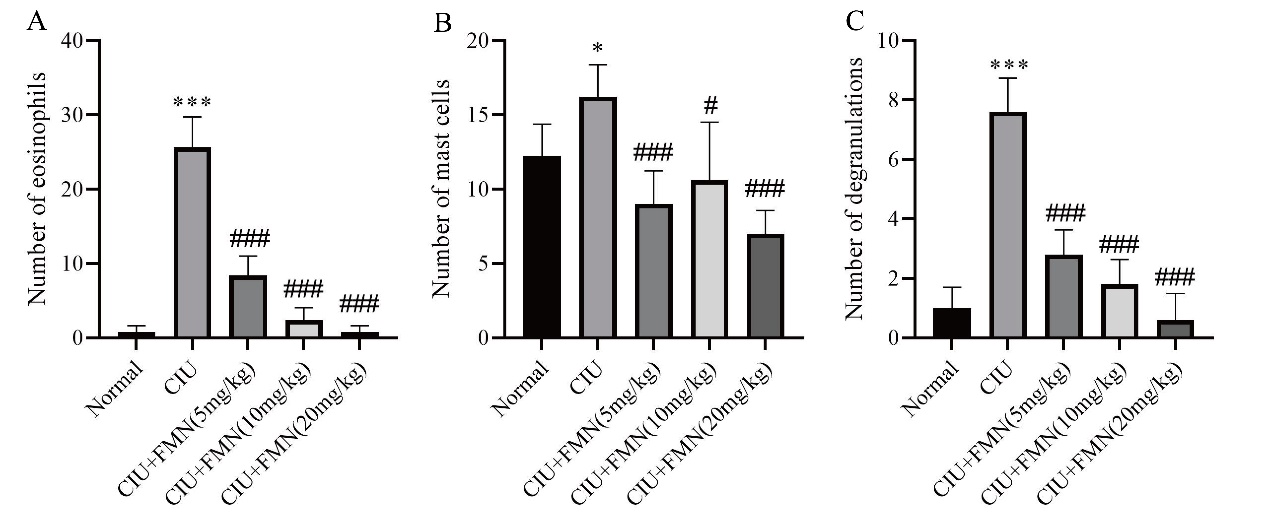


**Fig S1.** Formononetin ameliorates MCs degranulation and inflammation in CIU mice.

(A) The number of eosinophils per field, (B) the number of mast cells per field, and (C) the number of degranulated mast cells per field, corresponding to the histological images shown in Fig. 1B and Fig. 1C.

One-way ANOVA was used to determine significance in statistical comparisons. Statistical significance was defined at *p < 0.05, and *** p < 0.001 vs. the normal group; #p < 0.05, and ### p < 0.001 vs. the CIU model group. FMN, formononetin.
