# Supplementary figures and images for: Formononetin ameliorates SP-induced urticaria in mice via suppressing TAK1/MAK signaling pathway
Source: PLoS One. 2026 Jan 23;21(1):e0340078. doi: 10.1371/journal.pone.0340078 (PMC12829854; doi:10.1371/journal.pone.0340078)

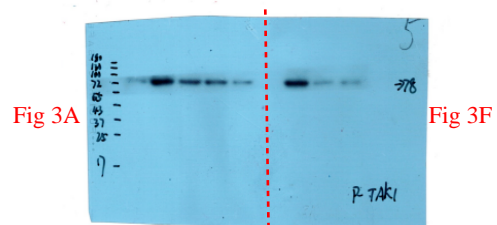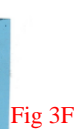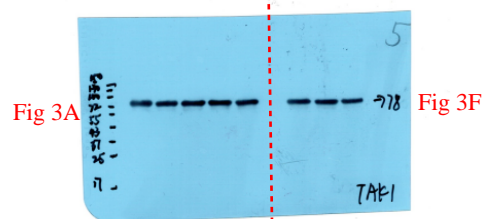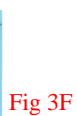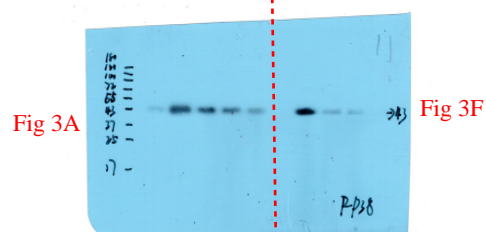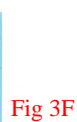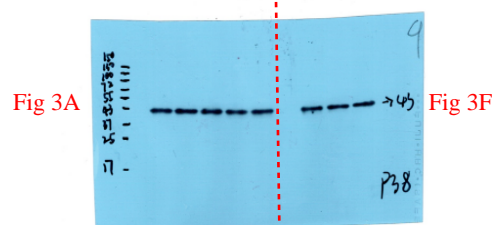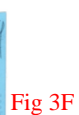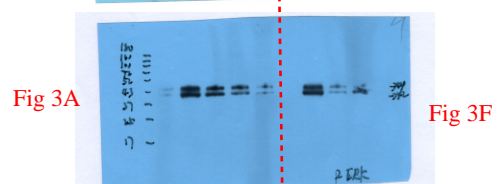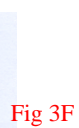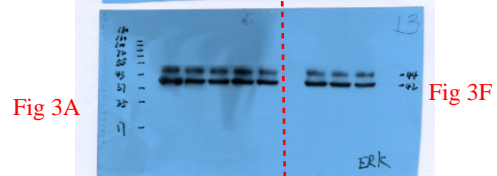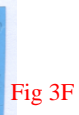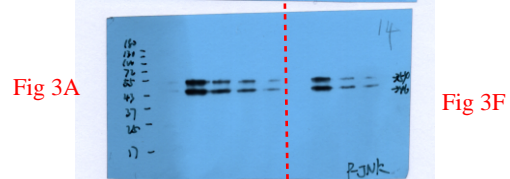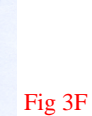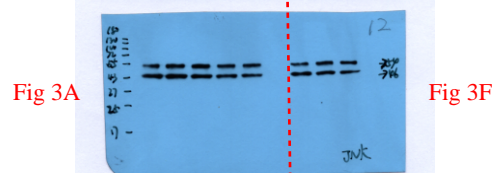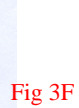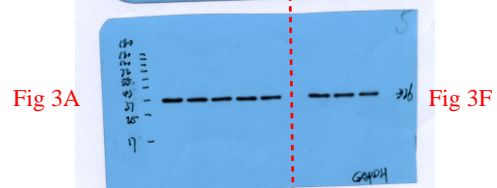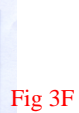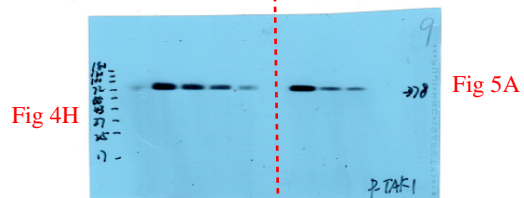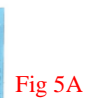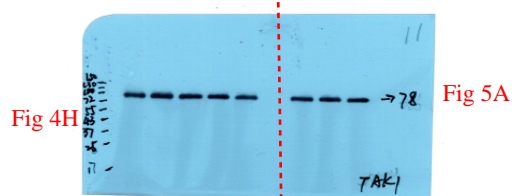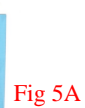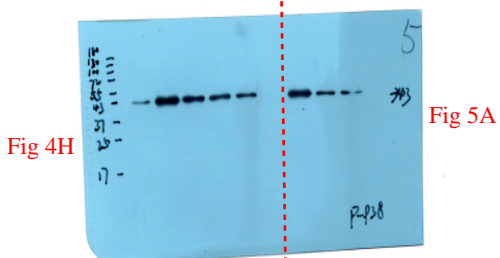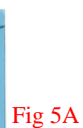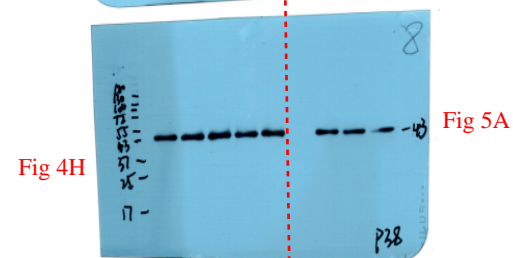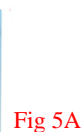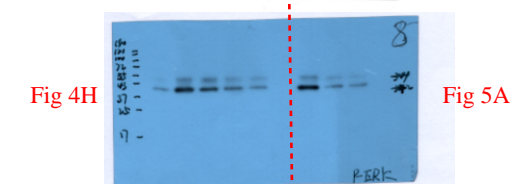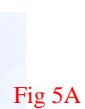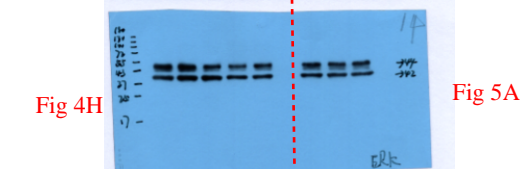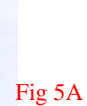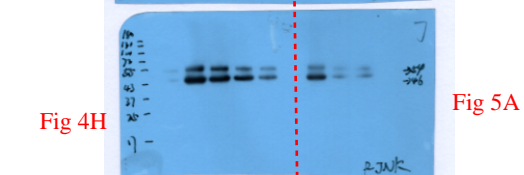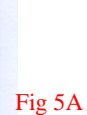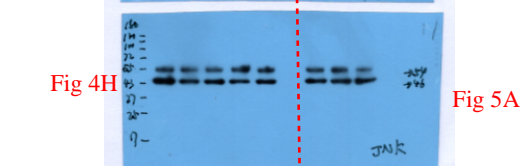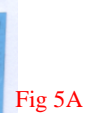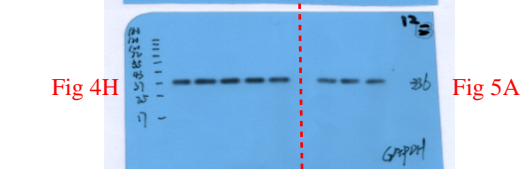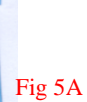

Supplement: S2 Fig — (PDF) [file pone.0340078.s002.pdf]
